# The Teaching of Medicine

**Published:** 1909-05-29

**Authors:** 


### The Hospital

A Journal of

The Medical Sciences and Hospital Administration.

New Series. No. 117, Vol. V. [No. 1189, Vol. XLVI.] SATURDAY, MAY 29, 1909.

### The Teaching of Medicine.

Slowly, and almost insensibly, the teaching of medical science has been undergoing revolution for many years. Gradually the ward has claimed more and more of the student's time, and the lecture-room less and less, while the clinical laboratory has also made heavy inroads upon his working hours. How far this decay of the lecture, as it may be called, is due to failure to maintain the old standard of lecturing, and how far to the inherent superiority of bedside training over any other, is not of any great moment; but probably most medical men of the younger generation will agree that the latter is the true explanation, for certainly there are lecturers to-day whose oratory it would be extremely difficult to surpass. The comparative values of the systematic lecture course, the ward clinic, and the study of text-books have often been discussed, and in view of the gradual decline in favour of the former during recent decades, the suggestions put forward by Dr. Byrom Bramwell* for the reform of the teaching system at Edinburgh are worthy of the most careful attention.

It would seem that in the Scottish capital the process of supersession, which has elsewhere overtaken the formal lecture, has made but little headway, and it is with the idea of bringing Edinburgh into line with less conservative schools that a committee of the Pathological Club has asked Dr. Bramwell for his ideas. The latter begins by laying down a series of propositions with which, speaking generally, we are in absolute agreement. He says the only systematic way of acquiring a satisfactory knowledge of medicine is by bedside study; lectures are far less valuable than study in the wards of a hospital. Edinburgh students attend too many lectures each day, and have far too little time for practical clinical work. The examination system in vogue encourages students to concentrate their energies far too much upon theoretical work and upon one subject at a time. This is the sum and substance of the objections urged against the prevailing methods of teaching medicine.

Now these propositions, which Dr. Bramwell evidently regards as incontrovertible, constitute in reality an indictment, not of the Edinburgh medical student, but of his educational authorities. If examinations are not sufficiently tests of a man's practical ability to diagnose and treat sick patients, the fault is among the examiners or the system under which they work. For Dr. Bramwell does not hesitate to affirm that cramming as opposed to sound clinical knowledge is the result of the present way of conducting the examinations. It is not surprising, therefore, to find among his suggestions the reform and rearrangement of the final examinations. To give students more time for the wards, Dr. Bramwell would reduce the course of systematic medicine lectures from 104 in two terms to 91 spread over the whole academic year, a modification which can scarcely be condemned as too sweeping. Apparently he would like to see a similar modification extended to the teaching of surgery. He would, further, abolish the clinical lectures on medicine (and on surgery), and would institute medical ward cliniques five days a week, in the hospital, instead of three, as at present; and he adds some criticisms of the tutorial clinical classes as at present conducted. Yet another suggestion is for the complete separation of gynæcology from clinical medicine and its association with obstetrics in a course to which he would allot 61 lectures annually. This step is also one which will surely commend itself to all but those with whom tradition is all powerful. It has always seemed to us that it is a mistake to rely entirely or mainly on internal examiners for university medical degrees, a tendency which, unless we err, is somewhat noticeable at Edinburgh. Dr. Bramwell does not offer any comments on the advantage of a fair sprinkling of external examiners, but the point is worth his consideration.

Upon the practicability of these alterations in the internal economics of the Edinburgh school we do not presume to speak; but if Dr. Bramwell's views upon the results of the present arrangements are correct, then, in principle, his remarks have our most cordial approbation. In passing it may be said that as Senior Physician to the Royal Infirmary, no less than by his distinguished record as a teacher and as a clinician, anything that Dr. Bramwell has to say on such a matter must be accorded the very weighiest consideration. The reduction in the number of lectures on medicine is not great, but it is

* *Edinburgh Medical Journal*, May, 1909.

at any rate enough to direct attention to the new conception of what a medical student ought to know, and where he ought to learn it. The old "signing-up" system is, in the present greatly altered status of the medical student, an anachronism. At a time when students had too little on their hands to occupy them fully, and in consequence were apt to grow slack in attendance, it was, no doubt, necessary in order to ensure progress that ordinances about the number of lectures to be attended should be enforced. But the detection of the ignoramus and his exclusion from the Medical Register are properly the affair of those who conduct examinations, not of teachers. There have always been, and always will be those who are impatient of lectures, especially if the lecturer be not of the most brilliant; such men get on far more quickly by the help of text-books and ward-work, and to handicap them by the old rules handed down from bygone generations, when text-books were few and poor, is a piece of folly as great as to allow the stolid youth who has slept through some hundreds of lectures credit for professional fitness. The signing-up system, in fact, is out of date, and it is high time to abolish it. Dr. Bramwell has many other suggestions, which are chiefly elaboration in detail of the broad lines first laid down, and consequently are of interest mainly to members of the Edinburgh school. Perhaps the most instructive is a paragraph dealing with the better distribution of students in the wards, and their limitation to 40 in any one ward—one per bed! If the congestion in the Royal Infirmary is really greater than this, it is hardly to be wondered at that clinical training is difficult to get, or that examiners are in some measure compelled to estimate the theoretical rather than the practical knowledge of those who present themselves for admission to the ranks of the profession. At the same time, it is a most remarkable tribute to the popularity of the Edinburgh school, and it shows that the teaching—even if, as Dr. Bramwell thinks, it is too theoretical—is thorough and attractive as far as it goes. The school is still the chief resort in Britain of colonial students; and in endeavouring to assimilate it to the spirit of the times, Dr. Bramwell and the Pathological Club will carry with them the sincerest well wishes of the medical profession.

